# *Bsal* susceptibility depends on host origin but not on skin microbiota in captive *Pleurodeles waltl.*

**DOI:** 10.1186/s42523-025-00485-x

**Published:** 2025-11-28

**Authors:** Léa Fieschi-Méric, Frank Pasmans, Eduardo Fernández Meléndez, Sofie De Bruyckere, Ellen Blomme, Elin Verbrugghe, An Martel,

**Affiliations:** Wildlife Health Ghent, Department of Pathobiology, Pharmacology and Zoological Medicine, Faculty of Veterinary Medicine, Ghent University, Salisburylaan 133, Merelbeke, B9820 Belgium

**Keywords:** Amphibia, *Batrachochytrium salamandrivorans*, Chytridiomycosis, Disease severity, Emerging infectious diseases, Microbiome, Microbiome volatility, Urodela

## Abstract

**Background:**

Amidst the current biodiversity crisis, amphibians are particularly endangered by the emergence of infectious diseases. The skin disease chytridiomycosis is caused by the fungi *Batrachochytrium dendrobatidis (Bd)* and *B. salamandrivorans (Bsal)*, which may interact with bacterial symbionts present on the amphibian epidermis. Extensive research has explored the interactions between the amphibian microbiota and *Bd*; yet, little is known about its interactions with *Bsal*. In this paper, we used the ribbed newt (*Pleurodeles waltl*), a model species displaying pronounced among-individual variation in response to *Bsal,* to (1) determine whether susceptibility to *Bsal* and individual microbiota vary between source groups; (2) test whether susceptibility to *Bsal* can be predicted from skin microbiota before exposure and (3) quantify microbiota volatility over time to determine whether *Bsal* infection intensity and chytridiomycosis severity correlate with the magnitude of shifts in bacterial communities caused by *Bsal* exposure.

**Results:**

Our results demonstrate that newts of different origin harbor distinct microbiota even under uniform rearing conditions. We show that *Bsal* infection intensity and disease severity cannot be predicted from the diversity, structure, or composition of the skin microbiota of *P. waltl.* Instead, a strong relation between newts’ source group and their response to *Bsal* suggests that other factors might underpin among-individual variation in *Bsal* susceptibility in this species. Moreover, our results indicate that the intensity of early *Bsal* infection and longer-term severity of chytridiomycosis do not correlate with the magnitude of microbiota change following *Bsal* exposure.

**Conclusion:**

These results demonstrate a limited involvement of the microbiota in *Bsal* dynamics in *P. waltl,* suggesting that other mechanisms contribute to individual *Bsal* susceptibility. Further research on the relation between chytrid pathogens and their amphibian hosts will be instrumental to improve the conservation of the most endangered vertebrate class on earth.

**Graphical Abstract:**

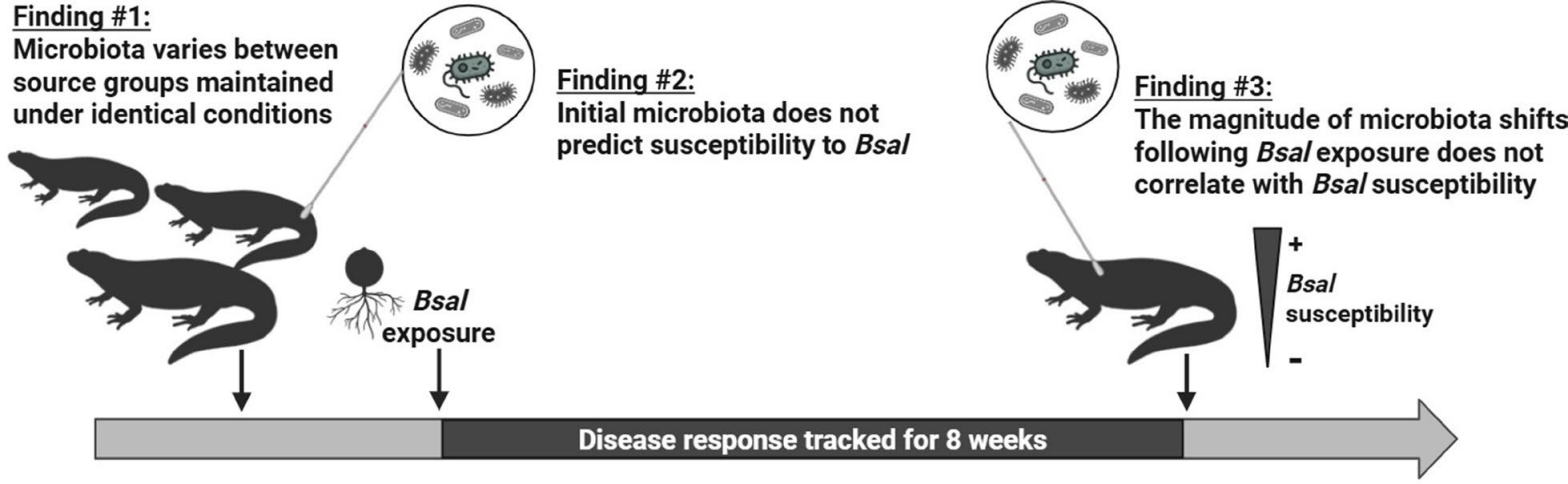

**Supplementary Information:**

The online version contains supplementary material available at 10.1186/s42523-025-00485-x.

## Background

Amphibians are currently the most endangered vertebrate class on earth, with over 40.7% of species globally threatened [1]. Multiple threats put them at risk of extinction, but the recent emergence of the infectious chytridiomycosis disease particularly aggravated their situation [2]. This disease is caused by two skin-infecting chytrid fungi: *Batrachochytrium dendrobatidis* (*Bd*), which led to the greatest recorded loss of vertebrate biodiversity attributable to a disease since its worldwide expansion in the 1990s [3], and *B. salamandrivorans* (*Bsal*) which recently spread from Asia to northwestern Europe, where it caused catastrophic declines among salamander populations [4, 5]. This latter fungus poses an unprecedented threat to urodeles [6–9] because of its high pathogenicity for a significant proportion of western Palearctic urodele species [10, 11].

*Bd* and *Bsal* zoospores invade, encyst and proliferate in the epidermis of susceptible amphibians, thereby disrupting its structure and vital functions (e.g., respiration, osmoregulation), eventually causing death in heavily infected individuals [12]. The amphibian skin and its mucus, including the communities of symbiotic micro-organisms referred to as ‘skin microbiota’, constitute the first line of defense against chytrid fungi [13]: in particular, some bacterial taxa can inhibit chytrid growth [14] and their presence on the epidermis can protect their host against chytridiomycosis [15–17]. Although microbiota are dynamic and their structure is constantly influenced by environmental and intrinsic factors, a core microbiota subset, characteristic of each individual, is generally stable [18] and is partially shared among members of the same species [19, 20]. Thus, amongst many other mechanisms [21], interspecific differences in composition of skin microbiota (particularly, the abundance of core chytrid-inhibitory taxa) can sometimes explain differences in susceptibility to chytrids between species [22, 23]. However, our understanding of the mechanisms behind within-species among-individual variation in response to chytrid exposure is limited. In the case of *Bd*, some studies have suggested that individual microbiota composition can predict infection outcomes [24], but most research finds no consistent relation between microbiota and differential susceptibility to *Bd* among individuals of the same species [25, 26]. In the case of *Bsal*, only one study investigated whether natural among-individual variation in microbiota structure (of the very susceptible *Triturus cristatus* and *Lissotriton vulgaris*) could predict subsequent *Bsal* burden (load) in vivo, and found no correlation between initial microbiota and later susceptibility [27]. Further research on other model species is needed to investigate whether variation in microbiota load, diversity and/or structure could underlie variation in susceptibility to *Bsal* between individuals.

More generally, factors causing among-individual variation in amphibian microbiota remain poorly characterized because studying the amphibian microbiota is particularly challenging. For example, the relative contribution of genetics in shaping different microbiota amongst wild populations cannot be distinguished from that of environmental variation [28–30]. While research on other taxa suggests that host genetics influence microbiota structure [31, 32], in amphibians, microbiota studies that include different populations are often conducted *in-situ*, making it difficult to disentangle environmental effects from host-driven factors [20]. Therefore, research conducted on amphibian populations maintained under uniform *ex-situ* conditions is necessary to deepen our understanding of among-individual variation in amphibian microbiota.

Beyond its potential role in infection resistance, the skin microbiome itself can be disrupted by pathogens. Exposure of amphibians to chytrid fungi generally causes dysbiosis, i.e., a disruption of the bacterial flora’s balance leading to a dysfunctional microbiota, through changes in the structure of the bacterial community (e.g., [24, 27, 33–35]) occasionally in conjunction with a reduction of its richness (e.g., [26, 36]), with deleterious consequences for the host [20]. Despite extensive research describing the effects of chytrid exposure on the amphibian microbiota, most studies have focused on *Bd*, and typically analyzed microbiota disturbance as a binary effect (by comparing control and exposed individuals) rather than considering continuous variation in microbiota structure. Only one study suggested that *Bd*-induced disturbance of the microbiota may be correlated with pathogen burden (*Bd* load) [25]. Whether the magnitude of *Bsal*-induced microbiota shifts could also be correlated with susceptibility to this chytrid remains unknown.

To fill these gaps in knowledge, this study investigates skin microbiota dynamics in the context of exposure to *Bsal*. Experimental infections were carried out on ribbed newts (*Pleurodeles waltl*), a relevant model species for this study since it displays among-individual variation in *Bsal* susceptibility. The newts were provided by different breeders, allowing us to investigate whether the differences in skin microbiota among source populations that are generally observed in wild amphibians [28–30], are also present in captive-bred newts despite uniform artificial rearing conditions. Non-invasive skin swabs were collected before and after chytrid exposure to characterize individual skin microbiota through 16S rRNA gene sequencing. In order to evaluate *Bsal* susceptibility in a quantifiable and holistic manner, two custom-built metrics were used: *Bsal* infection intensity (II), which measures early infection kinetics (rate of increase in *Bsal* load on a short timeframe following exposure), and *disease severity* (DS), which encompasses the full severity of the disease (longer-term effects on *Bsal* burden, infection kinetics, and body-condition). We explored (1) whether individuals coming from different source groups varied in their susceptibility to *Bsal* (II and DS) and in their microbiota; (2) whether *Bsal* susceptibility could be predicted from initial microbiota diversity, structure or composition; and (3) whether microbiota shifts following *Bsal* exposure were correlated with susceptibility. Overall, this study contributes to our understanding of the intricate relations between chytrids, microbes and their amphibian hosts, which is crucial for improving amphibian conservation.

## Methods

### Animal collection and experimental exposures

Forty-three adult captive-bred ribbed newts (*P. waltl*) provided by three different breeders were used in this study: Source group 1 (*n* = 20), Source group 2 (*n* = 12) and Source group 3 (*n* = 11; Supplementary Table 1). Upon their arrival in our facilities, all newts were housed per group and maintained under the same artificial rearing conditions. The experiment started (Day 0) with the collection of whole-body skin microbiota swab samples (MW100 rayon tipped dry swab, MWE, Corsham, UK) as described in Fieschi-Méric et al. [37]. Each individual was handled with a new pair of gloves to avoid cross-contamination. All newts were weighed, measured, and injected a passive integrated transponder (PIT-tag; AL-VET ID ISO MINI, Dechra) in their coelomic cavity. They were subsequently maintained in individual boxes and fed calcium- (Repti-Calcium with D3, Zoo Med) and vitamin-supplemented (Reptivite Vitamin D3, Zoo Med) crickets (*Acheta domestica*) and buffalo worms (*Alphitobius diaperinus*) *ad libitum.*

Once they had healed from the PIT-tag surgery (Day 77), all newts were exposed to *Bsal*. Each individual was swabbed weekly (CLASSIQSwabs 160C, COPAN) to quantify *Bsal* load. The experiment ended 8 weeks after exposure to *Bsal* (Day 133): the weight and length of each individual were measured again, and the newts were heat-treated at 25 °C for 10 days to eliminate the infection [38]. Since Source group 1 exhibited the highest variation in response to *Bsal* (cf. Results), this group was selected to test the assumption that microbiome changes correlate with host response. From these 20 animals, a ventral microbiota skin swab was collected (10 strokes back and forth on the belly) before the heat treatment. All swabs were preserved dry at −80 °C until DNA extraction.

### *Bsal* inoculation and quantification

*Bsal* exposure was carried out by bathing each newt in a Petri dish filled with 1 mL of 8.8 × 10^3^ fresh *Bsal* spores (type strain AMFP13/01) for 24 hours at 15 °C [4].

Fungal DNA was extracted from weekly-collected swabs using PrepMan® Ultra Sample Preparation (Thermo Fisher Scientific, Waltham, USA), and was quantified through quantitative real-time PCR (qPCR) following Blooi et al. [39]. Reactions were conducted in duplicate and included a non-template control (NTCs) and a 10-fold dilution series of positive controls from 10^3^ to 10^−1^ genomic equivalents (GE) per 5 µL in each qPCR plate. The software CFX Maestro v2.3 (Bio-Rad) was used to retrieve quantification cycle (Cq) data and calculate the starting quantity (SQ) of GE in the 5 µl of DNA used for each qPCR reaction. *Bsal* loads were averaged over duplicate reactions and expressed as log₁₀(GE per swab).

### *Bsal* susceptibility quantification: infection intensity and disease severity indices

*Bsal* load quantification through qPCR was used to generate individual pathogen load curves over time. Then, response to *Bsal* exposure was evaluated using two custom-built metrics: *infection intensity* (II), defined as the early response to *Bsal* exposure, and *disease severity* (DS), encompassing the full ramifications of the infection on a longer term. II was defined as the value of the maximal slope of the load curve (from null load before the exposure started, to peak *Bsal* load) within the first 4 weeks following exposure. DS was estimated over the 8 weeks following *Bsal* exposure using an index that included pathogen burden (total *Bsal* load), infection kinetics (latency from exposure to peak *Bsal* load), and body condition (body mass index: mass (g)/snout-vent length (mm)^2^) variation between the start and the end of the experiment (Box 1). The R code to build them is available at Figshare repository (https://figshare.com/s/d9ea685218fdc79a9b37).

#### Box 1

Build-up of the disease metrics.

**Fig. 1:**
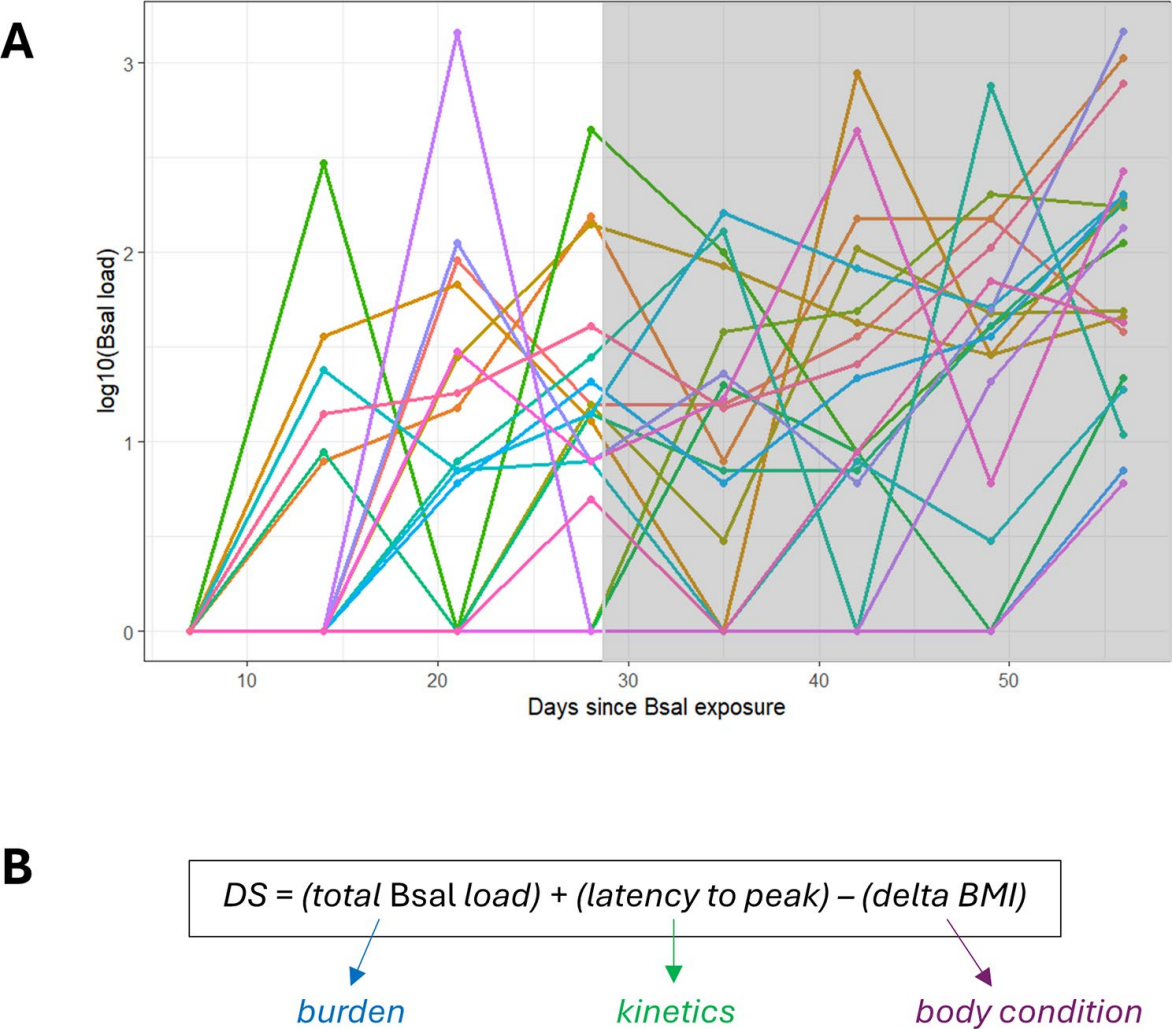
Individual curves of *Bsal* load in *P. waltl* from Population 1. Each individual is represented by a different color. The white area of the curves was used to compute the Infection Intensity metric (**A**). Formula used to build the Disease Severity index encompassing the full severity of the disease (**B**)

For each individual, **Infection Intensity (II)** was assessed as the slope to peak *Bsal* load within the first 4 weeks post-exposure (white area of the curves, early response to pathogen; Fig. 1A).

For each individual, **Disease Severity (DS)** was calculated as the sum of:

- its total *Bsal* load (pathogen burden), measured as the area under the curve of *Bsal* load drawn from weekly qPCRs, standardized over all individuals;
- the latency to reach its peak *Bsal* load (infection kinetics), measured as the number of days between exposure to *Bsal* and day of maximal *Bsal* load for that individual, standardized over all individuals;
- its body-mass index (BMI) change (body condition), measured as the change in BMI between the last and first day of the experiment, standardized over all individuals (Fig. 1B)

### Bacterial DNA extraction and skin microbiota load quantification

Microbial DNA was extracted from all swabs using the DNeasy PowerSoil Pro kit (QIAGEN, Hilden, Germany), following the manufacturer’s instructions and including 7 non-template controls (NTCs) and 4 replicates of a mock community of known composition (ZymoBIOMICS Microbial Community Standard, ZYMO research, Irvine, USA). This DNA extract was used to determine total bacterial load using qPCR, and for 16S rRNA gene sequencing.

Microbiota samples from Source group 1 only were used for bacterial load quantification. In-silico PCRs conducted with published primers on the SILVA 16S database [40] suggested that the forward (5’-ACT CCT ACG GGA GGC AGC AG-3’) and reverse (5’-TTA CCG CGG CTG CTG G-3’) primers designed by Clifford et al. [41] detected most of the representative bacterial groups from a representative dataset of salamander skin microbiota. Therefore, these primers were used in a 0.5 µM concentration for qPCR-based quantification of total bacterial load, with a SensiFAST™ SYBR® No-ROX Kit (Bioline, London, UK) on a CFX384 detection system (Bio-Rad, Hercules, USA). Amplification consisted of DNA pre-denaturation at 95 °C for 10 minutes, followed by 40 cycles of denaturation (95 °C for 30 seconds) and annealing (60 °C for 1 minute), finished with an extension step (stepwise increase of temperature to 95 °C at 0.5°C/5 seconds). Reactions were conducted in triplicate. Non-template controls (NTC) and a 10-fold dilution series of positive controls (DNA from *Clostridium perfringens*) from 10^7^ to 0 GE per 2 µL were also included in triplicate in each qPCR plate. The software CFX Maestro was used again to retrieve Cq data and calculate the SQ of 16S rRNA copies in the 2 µl of DNA used for each qPCR reaction. When Cq values varied by more than 0.5 between triplicates, the outlier reaction was discarded from the dataset. Following Vences et al. [42], average bacterial load per swab was estimated as: (average SQ per sample - average SQ of DNA extraction NTCs) * 25 (to account for the total amount of 50 µl DNA extraction yield per sample).

### Skin microbiota sequencing

Microbiota was sequenced from all swabs. Following the protocol outlined in Aguirre et al. [43], the V3–V4 hypervariable region of the 16S ribosomal RNA gene (~464 bp) was amplified using the S-D-Bact-0341-b-S-17 (5′-CCT ACG GGN GGC WGC AG-3′) and S-D-Bact-0785-a-A-21 (5′-GAC TAC HVG GGT ATC TAA TCC-3′) primers [40] flanked with Nextera adapters that included 2 phosphorothioate bonds on each end for increased stability against exonucleases. Amplicons were purified using CleanNGS beads (CleanNA, Waddinxveen, The Netherlands) before a second PCR was conducted to attach dual indices and flow-cell binding sequences to the amplicons (Nextera XT Index Kit, Illumina, San Diego, USA). The final PCR products were purified using CleanNGS beads again. Purified barcoded libraries were then quantified using a Quantus double-stranded DNA assay (Promega, Madison, USA) before being combined into an equimolar 10 nM pool, and sequenced on an Illumina MiSeq system (Illumina, San Diego, USA) at a depth of 70,000 reads per sample (2 × 300 bp, paired-end) by Macrogen (Seoul, South Korea). Swabs collected from all three source groups before chytrid exposure (*n* = 43) were processed in a different sequencing run than post-exposure swabs collected solely from Source group 1 (*n* = 20).

### Bioinformatics

All sequencing data generated for the current study is available in the NCBI Sequence Read Archive (BioProject ID: PRJNA1248485). Demultiplexed sequences were preprocessed using DADA2 v.1.30.0 [44]. Forward and reverse reads were truncated at positions of decreasing quality (respectively 280 and 250 bp cycles), and chimeric Amplicon Sequence Variants (ASVs) were removed by reconstruction against more abundant parent ASVs on the merged dataset from both sequencing runs. Taxonomy was assigned to representative sequences using the naïve Bayesian classifier implemented in DADA2 and the SILVA reference database [45]. Alignments at 97% sequence identity were accepted up to the genus level, but only exact-matching was used for species-level assignment [46]. In addition, representative sequences were aligned to the database of enhancing and inhibitory isolates from Bletz et al. [33]: none of the ASVs in our dataset matched phylotypes with known activity against *Bsal* in that database.

Processing of the sequences was carried out using the R package phyloseq [47]. Only bacterial sequences were kept, and 25 contaminant ASVs identified from NTCs were removed using the R package decontam [48]. Spurious ASVs present in less than 5% of samples were filtered out [49], and because the depth of coverage varied by an 11-fold between samples, library sizes were normalized to 30,000 reads using rarefaction without replacement [50]. The filtered and normalized dataset comprised 1,176 ASVs across a total of 63 samples from 43 different newts, while the raw (unfiltered and not normalized) dataset was comprised of 3,239 ASVs. Raw data was used to compute differential abundance analyses and alpha-diversity (within-sample diversity) Chao1 (estimated ASV richness) and Shannon (estimated ASV evenness) metrics. The filtered and normalized data was used to measure beta-diversity with weighted Unifrac distances (phylogenetic distance weighted by species abundance information) to investigate differences in community structure among samples [51].

### Statistical analysis

Analyses were conducted in the R environment v.4.4.2 [52]; all data and code are publicly available at Figshare repository (https://figshare.com/s/d9ea685218fdc79a9b37). Statistical tests were deemed significant if associated with *p-values* below a 0.05 threshold (and below a 0.01 threshold for differential abundance analyses).

First, we investigated whether the three source groups differed in their microbiota and/or response to *Bsal*. To this end, several analyses of variance (ANOVAs) were carried out, with source group as the explanatory variable, and microbiota alpha-diversity (Chao1 or Shannon) or *Bsal* susceptibility (II or DSI) as the response variable. When these tests indicated significant average differences between source groups, they were followed by pairwise t-tests and *p-values* were adjusted for multiple comparisons using the Benjamini-Hochberg method (*p-adj*). When the assumption of homoscedasticity associated with ANOVAs was violated, a Welch one-way test was used instead. Differences in among-individual variance (homoscedasticity) between source groups for each of the response variables were explored using Barlett tests, followed by pairwise F-tests. To determine whether microbiota structure (beta-diversity) differed between source groups, permutational multivariate analyses of variance (PERMANOVAs) implemented with the adonis function (*n* = 9,999 permutations) were used, followed by pairwise adonis tests. Among-individual variation in beta-diversity was compared across source groups using a betadisper test. A differential abundance analysis was carried out on raw data using DEseq2 [53] to investigate differences in microbiota composition between source groups.

Secondly, we tested whether microbiota before *Bsal* exposure could predict subsequent response to the chytrid. To this end, linear mixed-models were built with *Bsal* susceptibility (II or DSI) as the response variable and initial (Day 0) microbiota alpha-diversity (Chao1 or Shannon) as the explanatory variable. Since the preceding analysis revealed significant differences among source groups in their microbiota composition, diversity and structure, and in their *Bsal* susceptibility, source group was included as a random effect in these linear mixed-models. The *p*-values associated with each covariate were calculated using Satterthwaite’s approximation. To determine if the microbiota of newts with different susceptibility to *Bsal* initially varied in beta-diversity, PERMANOVAs stratified by source group were used.

Finally, we explored whether the breadth of microbiota change following *Bsal*-exposure was correlated with the magnitude of individual response to this pathogen. This part of the study focused on Source group 1 since the first analysis revealed that this group was associated with the highest among-individual variability in *Bsal* susceptibility, as well as in microbiota diversity. Pearson correlations were used to investigate whether temporal changes (delta Day 133 – Day 0) in microbiota load or alpha-diversity (Chao1 or Shannon) were correlated with *Bsal* susceptibility (II or DSI). When assumptions of normality associated with Pearson correlations were violated, a Kendall correlation test was used instead. A volatility analysis was carried out to determine if the magnitude of the structural change of the microbiota (beta-diversity) was correlated with the magnitude of the response to *Bsal* (II or DSI). Briefly, we calculated individual volatility as the Aitchison distance between microbial communities at Day 133 and Day 0, following Bastiaanssen et al. [54]. We used Pearson correlations to investigate the relation between microbiota volatility and *Bsal* susceptibility.

## Results

### Newt source group determines *Bsal *susceptibility and microbiota composition, diversity and structure

**Fig. 2:**
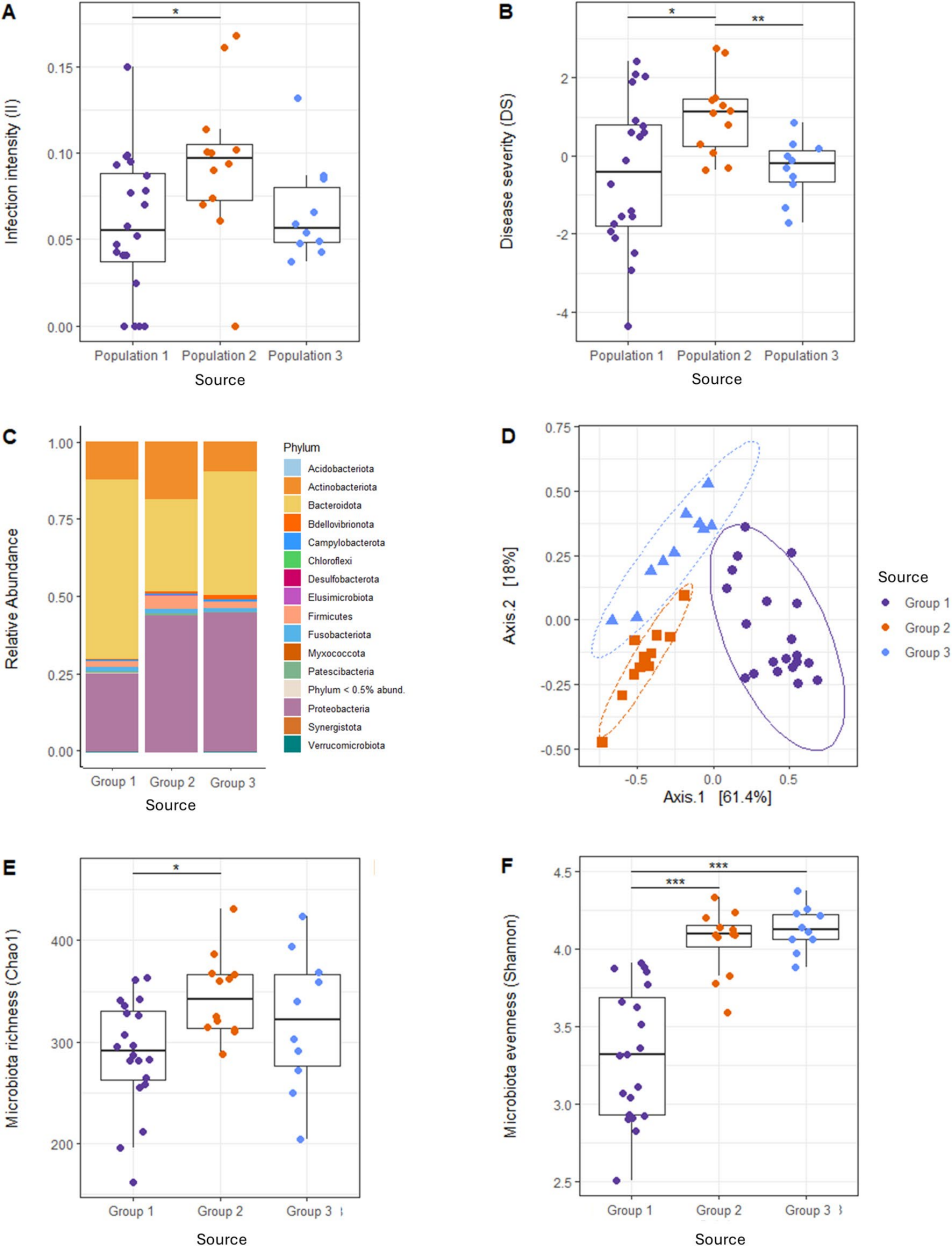
Susceptibility to *Bsal* and skin microbiota vary between source groups. Effect of the group of origin on *Bsal* infection intensity (**A**) and chytridiomycosis severity (**B**). Relative abundance of the major bacteria phyla in the initial (day 0) skin microbiota of newts from different origins (**C**). PCoA plot representing the initial beta-diversity of the microbiota of newts from different origins, measured with weighted unifrac distances (**D**). Effect of the group of origin on initial skin microbiota alpha-diversity, measured with the Chao1 (**E**) and the Shannon (**F**) indices

*Bsal* susceptibility differed between the three source groups, both in terms of average II (*F*_(*2,39)*_ = 3.37, *p-value* = 0.045) and DS (*F*_*(2,26)*_ = 7.36, *p-value* = 0.003). More precisely, average II was significantly lower in individuals from Source group 1 compared to Source group 2 (*p-adj* = 0.043), but was not different between the other groups (Source groups 1–3: *p-adj* = 0.589; Source groups 2–3: *p-adj* = 0.146), and average DS was significantly higher in individuals from Source group 2 compared to Source group 1 (*p-adj* = 0.011) and to Source group 3 (*p-adj* = 0.005), but was not different between individuals from Source groups 1 and 3 (*p-adj* = 0.818). Among-individual variability in II was similar across source groups (*K*^*2*^ = 1.81, *df* = 2, *p-value* = 0.405) whereas the variance in DS differed (*K*^*2*^ = 10.20, *df* = 2, *p-value* = 0.006): DS varied significantly more amongst newts from Source group 1 compared to Source group 2 (*F*_*(19,11)*_ = 3.52, *p-value* = 0.037) and to Source group 3 (*F*_*(19,9)*_ = 6.16, *p-value* = 0.008) but was not different between Source groups 2 and 3 (*F*_*(11,9)*_ = 1.75, *p-value* = 0.411; Fig. 2A–B).

Before exposure to *Bsal*, the skin microbiota of the newts was overall dominated by Bacteroidota (45%), Proteobacteria (35%) and Actinobacteria (13%). However, in contrast with Source group 1, the microbiota of Source groups 2 and 3 comprised relatively more Proteobacteria than Bacteroidota (Fig. 2C). Differential abundance analyses indicated that hundreds of phylotypes differed in abundance between each population (Supplementary Tables 2–4). Initial microbiota structure was significantly different between newts of different origin (*F*_(*2,39*)_ = 32.73, *R*^*2*^ = 0.63, *p-value* < 0.001; Supplementary Table 5), but among-individual variation in beta-diversity was homogenous across source groups (*F*_*(2,39*)_ = 0.55, *p-value* = 0.581; Fig. 2D). Microbiota diversity was significantly different between source groups (Chao1: *F*_*(2,39*)_ = 4.18, *p-value* = 0.023; Shannon: *F*_*(2,25*)_ = 29.52, *p-value* < 0.001): the average microbiota richness was lower in newts from Source group 1 compared to Source group 2 (*p-adj* = 0.021) but was not different between the other groups (Source groups 1–3: *p-adj* = 0.208; Source groups 2–3: *p-adj* = 0.298), and microbiota evenness was significantly lower in individuals from Source group 1 compared to Source group 2 (*p-adj* < 0.001) and to Source group 3 (*p-adj* < 0.001), but not between newts from Source groups 2 and 3 (*p-adj* = 0.300). Among-individual variability in microbiota richness was similar across newts of different origin (*K*^*2*^ = 2.59, *df* = 2, *p-value* = 0.275) whereas variance in microbiota evenness differed (*K*^*2*^ = 13.36, *df* = 2, *p-value* < 0.001): alpha-diversity measured with the Shannon index varied significantly more across newts from Source group 1 compared to Source group 2 (*F*_*(19,11)*_ = 4.04, *p-value* = 0.022) and Source group 3 (*F*_*(19,9*)_ = 8.76, *p-value* = 0.002) but did not differ between Source groups 2 and 3 (*F*_*(11,9*)_ = 2.17, *p-value* = 0.255; Fig. 2E–F).

### Microbiota does not predict *Bsal* susceptibility

**Fig. 3:**
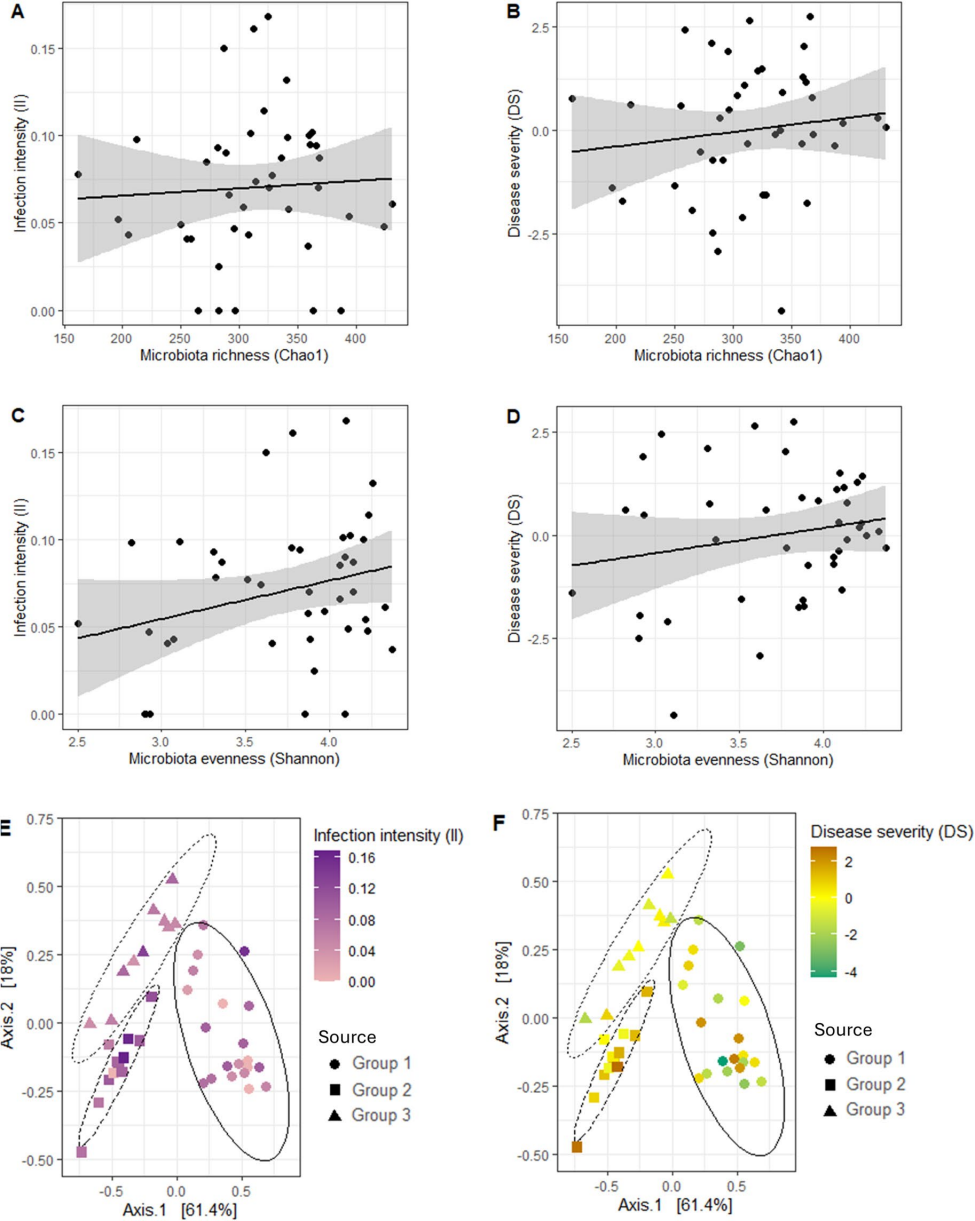
Salamander skin microbiota does not predict susceptibility to *Bsal*. Relation between initial (day 0) skin microbiota richness measured with the Chao1 index and later (day 133) infection intensity (**A**) and disease severity (**B**). Relation between initial skin microbiota evenness measured with the Shannon index and later infection intensity (**C**) and disease severity (**D**). PCoA plot representing the initial beta-diversity of the microbiota, measured with weighted unifrac distances, of individuals with varying infection intensity (**E**) and disease severity (**F**) following *Bsal* exposure. Color gradients represent *Bsal* susceptibility and the shape of the datapoints indicate the group of origin of the newts

Across the three source groups, II could neither be predicted from initial microbiota richness (*β* = −4.59e-05, *df* = 40, *p-value* = 0.688) nor from initial microbiota evenness (*β* = 0.02, *df* = 9.93, *p-value* = 0.293; Figs. 3A, 3C). Similarly, microbiota diversity before *Bsal* exposure was not a reliable predictor of DS (Chao1: *β* = 2.86e-04, *df* = 40, *p-value* = 0.947; Shannon: *β* = 0.22, *df* = 18.91, *p-value* = 0.730; Figs. 3B, 3D). Microbiota structure before exposure to *Bsal* was neither related to II (*F*_(*1,40)*_ = 2.09, *R*^*2*^ = 0.05, *p-value* = 0.788) nor DS (*F*_*(1,40)*_ = 3.18, *R*^*2*^ = 0.07, *p-value* = 0.392; Fig. 3E–F).

### The magnitude of microbiota change following *Bsal* exposure is neither correlated to infection intensity nor to chytridiomycosis severity

**Fig. 4:**
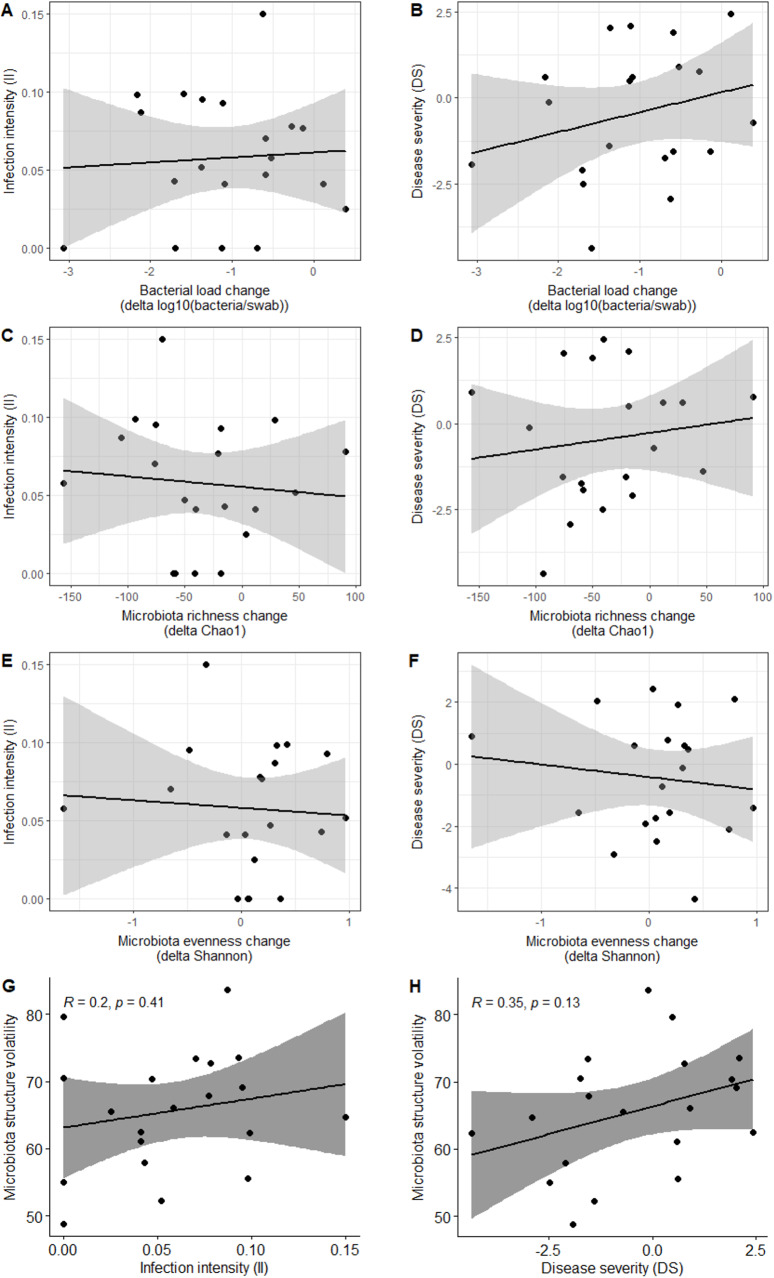
The magnitude of microbiota volatility following *Bsal*-exposure is not correlated with the intensity of *Bsal* infection nor with the severity of chytridiomycosis disease. Relation between the change in epidermal bacterial load following *Bsal* exposure (delta Day133 - day 0) and infection intensity (**A**) and disease severity (**B**). Relation between the change in microbiota richness following *Bsal* exposure (delta Chao1) and infection intensity (**C**) and disease severity (**D**). Relation between the change in microbiota evenness following *Bsal* exposure (delta shannon) and infection intensity (**E**) and disease severity (**F**). Relation between microbiota volatility following *Bsal* exposure (delta beta-diversity) and infection intensity (**G**) and disease severity (**H**)

Over the course of the experiment, skin microbiota load and richness tended to decrease but microbiota evenness slightly increased (Fig. 4A-F). The intensity of *Bsal* infection (II) was neither correlated with the magnitude of within-individual change in skin bacterial load (*t* = 0.28, *df* = 18, *p-value* = 0.781; Fig. 4A), nor with the magnitude of within-individual change in microbiota diversity (delta Chao1: *t* = −0.40, *df* = 18, *p-value* = 0.697; delta Shannon: *z* = 0.62, *p-value* = 0.536; Fig. 4C, E). DS was not correlated with the magnitude of within-individual change in skin bacterial load (*t* = 1.13, *df* = 18, *p-value* = 0.273; Fig. 4B), nor with the magnitude of within-individual change in microbiota diversity (delta Chao1: *t* = 0.61, *df* = 18, *p-value* = 0.549; delta Shannon: т = 93, *p-value* = 0.924; Fig. 4D, F). Microbiota structure volatility was not significantly correlated with *Bsal* susceptibility despite a positive trend with II (*t* = 0.85, *R* = 0.20, *df* = 18, *p-value* = 0.407) and with DS (*t* = 1.59, *R* = 0.35, *df* = 18, *p-value* = 0.130; Fig. 4G–H).

## Discussion

### The resident skin microbiota of ribbed newts plays a minor role in *Bsal* susceptibility

Our results show that *Bsal* susceptibility cannot be predicted from pre-exposure individual microbiota in *P. waltl*. This finding is consistent with the only other study that investigated whether microbiota structure could predict subsequent *Bsal* burden, to our knowledge [27]. However, it should be noted that a shorter timeframe between microbiota sampling and *Bsal* exposure than the one used in our study could have captured microbiota dynamics more precisely; indeed, individual microbiota may have changed between our initial skin swabbing event and the time of infection with *Bsal*. The delay between our initial microbiota sampling and *Bsal* exposure was set to allow the microbiota to recover its normal state after the PIT-tag surgery, since we know that animal microbiota reversibly change during wound healing [55]. Although microbiota are generally stable in adult amphibians maintained in captivity [37], we cannot exclude that time-related changes in the microbiota of our newts could have naturally occurred over that delay.

Our results also suggest that microbiota volatility following *Bsal* exposure is not significantly correlated with II nor with DS. We cannot exclude that with a bigger sample size, and stronger among-individual variation in microbiota structure, the unsignificant positive trend between microbiota volatility and DS may have become significant. Indeed, our experimental newts were captive-born and had been maintained together in the same terraria per source group before the experiment, two phenomena which are known to severely reduce the alpha- and beta-diversity of animal microbiota, respectively [37, 56, 57]. Moreover, the only studies reporting a link between microbiota and *Bd*-susceptibility were conducted in the wild [24], or using a realistic field-like setting [25]. The absence of bacteria with known *Bsal*-inhibitory activity in our dataset could result from the impoverishment of amphibian microbiota in captivity [37], or from the very limited number of *Bsal*-assays available in the literature (with only one database of *Bsal*-inhibitory bacteria to our knowledge [33]; ). Thus, research conducted on wild amphibians, which generally have more diverse microbiota than captive individuals, will be fundamental to further investigate the relation between bacterial communities and susceptibility of their hosts to *Bsal*.

The slight increase of microbiota evenness, and decrease of microbiota load and richness over the course of the experiment suggest that some bacterial taxa were lost but that the remaining taxa were more evenly distributed following *Bsal* exposure. Previous research reported that *Bsal* infection can result in subtle increases in the relative abundance of opportunistic bacteria responsible for the septicemic events associated with chytridiomycosis [33], but the absence of a strong relation between *Bsal* susceptibility and microbiota volatility following *Bsal* exposure suggests again that skin bacterial communities have a limited role in *Bsal*-induced chytridiomycosis dynamics. This contrasts with *Bd*, since in some species, susceptibility to *Bd* can be predicted from differences in microbiota composition [24] and the magnitude of *Bd*-induced disturbance of the microbiota can be correlated with pathogen burden [25]. Overall, this suggests a potentially different involvement of the amphibian microbiota in chytridiomycosis dynamics across its etiologic agents, with a stronger and more direct link between *Bd* and host skin bacterial communities, compared to *Bsal.* Thus, other mechanisms may strongly contribute to *Bsal* susceptibility.

With only three source groups, our experimental set-up did not allow to test whether this absence of relation between individual microbiota and susceptibility phenotype extended to the group-level, i.e., whether average microbiota variation may coincide with differences in mean *Bsal* susceptibility across source groups. Given the observed variation between source groups in our study, we strongly encourage future research on amphibian disease susceptibility and/or microbiota to include several populations in their design in order to further investigate interactions at this level. Moreover, although our volatility analysis yielded unsignificant results, we wish to emphasize the usefulness of this recent approach [54]. Indeed, microbiota dysbiosis may be continuous rather than a simplistic binary process; in this context, volatility analyses allow to study variations of microbiota structure along gradients and timelines. The field of study of the amphibian microbiota would therefore benefit from a paradigm shift with the adoption of volatility analyses to investigate microbiota variation.

### Beyond the microbiota: wider interactions within the amphibian skin ecosystem may underpin *Bsal* susceptibility

The three source groups included in this study significantly differed in their microbiota composition, diversity and structure. Such conserved bacterial signature of their origin was unexpected given that within captive populations, skin microbiota are typically low in diversity and very similar among individuals because of the lack of environmental sources of microorganisms [27; 37]. Extensive literature describes the existence of population-level variation in the microbiota of wild amphibians, but the degree to which environmental differences in the niches occupied by these populations underpin this variation could hardly be determined [28–30]. The observed differences among our captive source groups maintained under identical conditions highlight the fact that amphibian microbiota are not simply a reflection of the available bacteria in their environment [19]. Several mechanisms may underpin microbiota assemblages on the amphibian skin, such as maternal or developmental effects [58; 59]. In addition, differences in feeding regimes between the three source groups before their arrival in our facilities could have had a lasting influence on their skin microbiota [60]. Given that in some frog species, genetic distance among individuals correlates with their associated microbial community dissimilarity [28], the observed microbiota variation in our study could also result from putative genetic differences between source groups. Such genetic basis of a core microbiota would explain the observed persistence of a unique population-signature in the microbial assemblages of amphibians translocated between similar environments [61, 62].

The significant variation in *Bsal* susceptibility across source groups in our study further suggests the existence of genetic, epigenetic, developmental and/or maternal effects. Carry-over effects from potentially different breeding conditions before arrival in our facilities could have affected disease susceptibility [63]. Differences in immune-related gene expression or maternal transmission of immune factors [64–66] could also have affected *Bsal* susceptibility and microbial assemblages simultaneously. For example, the secretion of antimicrobial peptides with chytrid-inhibitory activity [67, 68] may vary among individuals, resulting in different responses to chytridiomycosis, as well as different microbiota composition. Species-specific variation in skin secretions of anti-fungal peptides can explain interspecific differences in chytridiomycosis susceptibility [69, 70], but little is known about within-species among-individual variation in secretion patterns [71]. Moreover, differences in skin structure, notably in membrane receptors that may interact with *Bsal*, could underpin differences in susceptibility among individuals. Indeed, a recent study showed that interspecific variation in susceptibility to *Bsal* was correlated with differences in skin structure, notably in epidermal glycosylation patterns, between species [72]. Thus, individual differences in the expression of glycosylation-related genes could explain among-individual variation in *Bsal* susceptibility in *P. waltl.* In addition, these epidermal structures might constitute fundamental substrates not only for *Bsal*, but also for the adhesion of symbiotic bacteria on the skin, thus explaining differences in microbiota between source groups.

Taken together, these new insights highlight the importance of studying further the interactions within the amphibian skin ecosystem, notably by using a comprehensive lens that integrates gene expression, microbiota and chytrids, amongst other candidates. The adoption of such a holistic approach could open new perspectives for amphibian conservation, by contributing to the development of bioaugmentation or selective breeding strategies, for example.

## Conclusions

This study demonstrates a strong relation between *Bsal* susceptibility and the origin of the newts, suggesting that genetic, developmental, or maternal effects may be strong contributors to individual variation in *Bsal* susceptibility in *P. waltl.* Moreover, our results show that individual *Bsal* susceptibility cannot be predicted from microbiota in that species, and that the magnitude of microbiota change following *Bsal* exposure is not correlated with *Bsal* infection intensity nor with chytridiomycosis severity. These findings indicate a limited involvement of the microbiota in *Bsal* dynamics, further suggesting that other mechanisms may underpin individual differences in *Bsal* susceptibility. Future research investigating genetic expression in differentially susceptible individuals will be instrumental to further our understanding of *Bsal* resistance mechanisms, and more generally to improve the conservation of the most endangered vertebrate class on earth.

## Electronic supplementary material

Below is the link to the electronic supplementary material.

Supplementary Material 1

Supplementary Material 2

Supplementary Material 3

Supplementary Material 4

Supplementary Material 5

Supplementary Material 6

## Data Availability

All sequencing data generated for the current study is available in the NCBI Sequence Read Archive (BioProject ID: PRJNA1248485) and the R code generated to analyze the data can be found at Figshare repository (https://figshare.com/s/d9ea685218fdc79a9b37).

## Abbreviations

ASV: Amplicon Sequence Variant
*Bsal*: *Batrachochytrium salamandrivorans*
*Bd*: *Batrachochytrium dendrobatidis*
Cq: quantification cycle
DS: Disease severity
II: Infection intensity
NTC: Non-template control
PIT: Passive integrated transponder
SQ: Starting quantity
