# Supplementary material for: Bsal susceptibility depends on host origin but not on skin microbiota in captive Pleurodeles waltl
Source: Anim Microbiome. 2025 Nov 28;7:123. doi: 10.1186/s42523-025-00485-x (PMC12661705; doi:10.1186/s42523-025-00485-x)
Supplement: Supplementary file 6 — Supplementary Material 6 [file 42523_2025_485_MOESM6_ESM.docx]

**List of additional files**

**Supplementary Table 1.** Information about the newts used in this study.

Newts were provided by 3 different breeders (Origin). Sex could not be determined for some individuals (NA). Weight, length and BMI were measured at the start (Day 0) and the end (Day 133) of the experiment. Custom-built metrics were used to measure *Bsal* susceptibility (infection intensity, disease severity).

**Supplementary Table 2.** List of differentially abundant phylotypes in the initial microbiota of newts from Source group 2 compared to Source group 1.

The name used to identify each phylotype (ASV_reference) refers to the ASV table provided with the raw data on Figshare. The log2 fold change column indicates the magnitude of the difference in abundance of each taxa between Source group 2 and Source group 1 individuals, and the baseMean column indicates the direction of that change.

**Supplementary Table 3.** List of differentially abundant phylotypes in the initial microbiota of newts from Source group 3 compared to Source group 1.

The name used to identify each phylotype (ASV_reference) refers to the ASV table provided with the raw data on Figshare. The log2 fold change column indicates the magnitude of the difference in abundance of each taxa between Source group 3 and Source group 1 individuals, and the baseMean column indicates the direction of that change.

**Supplementary Table 4.** List of differentially abundant phylotypes in the initial microbiota of newts from Source group 2 compared to Source group 3.

The name used to identify each phylotype (ASV_reference) refers to the ASV table provided with the raw data on Figshare. The log2 fold change column indicates the magnitude of the difference in abundance of each taxa between Source group 2 and Source group 3 individuals, and the baseMean column indicates the direction of that change.

**Supplementary Table 5.** Results from pairwise adonis tests comparing the beta-diversity of the initial microbiota of the 3 source groups.

The contrast column indicates which source groups were compared, with the associated F statistic, R², degrees of freedom and p-value.


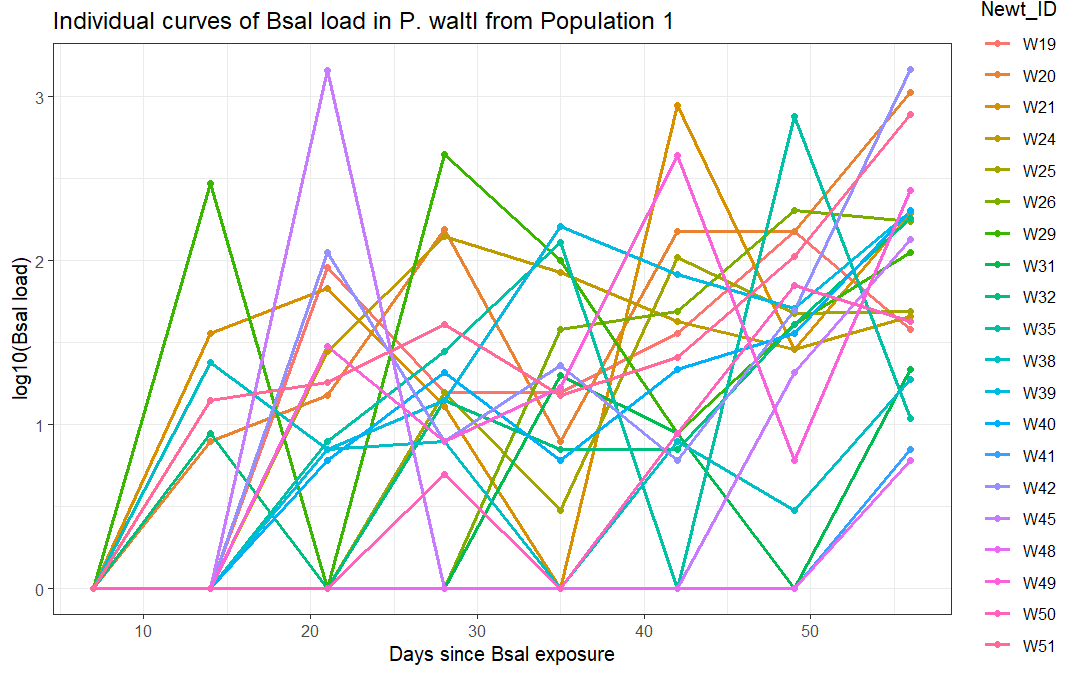


**Supplementary Figure 1.** Individual curves of *Bsal* load in *P. waltl* from Population 1. Each individual is represented by a different color. The white area of the curves was used to compute the Infection Intensity metric.
